# Activity of first-line epirubicin and paclitaxel in metastatic breast cancer is independent of type of adjuvant therapy

**DOI:** 10.1038/sj.bjc.6601634

**Published:** 2004-03-02

**Authors:** A Gennari, P Bruzzi, C Orlandini, B Salvadori, S Donati, E Landucci, V Guarneri, M Rondini, S Ricci, P Conte

**Affiliations:** 1 Department of Oncology, Division of Medical Oncology, Santa Chiara University Hospital, Via Roma 5756126, Pisa, Italy; 2 National Cancer Research Institute, Largo R. Benzi 1016132, Genoa, Italy; 3 Department of Oncology and Hematology, Division of Medical Oncology, University of Modena and Reggio Emilia, Via del Pozzo 71, 41100, Modena, Italy

**Keywords:** metastatic breast cancer, epirubicin, paclitaxel, adjuvant anthracyclines

## Abstract

To evaluate the impact of prior adjuvant chemotherapy on response rate (RR), progression-free (PFS) and overall survival (OS) of metastatic breast cancer patients treated with epirubicin/paclitaxel (ET) regimens. In all, 291 patients enrolled in five studies in metastatic breast cancer were analysed: 101 (35%) were chemonaive, 109 (37%) had received adjuvant CMF and 81 (28%) adjuvant anthracyclines. Response rate to ET was 66%. Response rate was 63% for cyclophosphamide plus methotrexate plus 5-fluorouracil (CMF), 67% for prior anthracyclines and 68% in chemonaive patients (*P*=0.5). By multivariate analysis, adjusted odds ratio for response was 0.81 (95% CI: 0.37–1.79) for CMF and 0.92 (95% CI 0.43–2.01) for anthracyclines (*P*=0.86). The CR rates were 14% for both CMF and anthracyclines and 22% for chemonaive patients (*P*=0.2). By multivariate analysis, the relative odds of CR for CMF or anthracyclines were 0.40 and 0.39 as compared to chemonaive patients (*P*=0.036). The median PFS was 11.0 months for prior CMF, 10.2 months for anthracyclines and 12.5 months in chemonaive patients (*P*=0.33). In multivariate Cox's model, a nonsignificant increase in the risk of progression was seen in patients treated with adjuvant CMF or anthracyclines. The median OS was 23.8 months for CMF, 20.2 months for anthracyclines and 27.5 months in chemonaive patients (*P*=0.61). The same, nonsignificant, association was seen in multivariate analysis. The ET regimens provide satisfactory results in metastatic breast cancer, regardless of previous adjuvant chemotherapy.

Metastatic breast cancer population is represented by a heterogeneous group of patients, and the objectives of therapy range from symptom palliation and minimisation of toxicity in elderly women with indolent disease to prolongation of survival in younger women, with good performance status and aggressive visceral disease. In particular, for patients with receptor-negative disease, or for those whose disease has become resistant to endocrine therapy and in whom impending organ failure requires a rapid response, cytotoxic chemotherapy with the most active drugs is generally the first treatment option to be considered.

The administration of anthracyclines was associated with an increase in response rate (RR), remission duration and survival of patients with metastatic disease, with approximately 20% of complete responders still disease free 10 years after achieving a complete response (CR) (Falkson *et al*, 1995; Rahman *et al*, 1999). Moreover, an overview of randomized studies evaluating first-line chemotherapy in metastatic disease showed that polychemotherapy yields a higher RR on average than single-agent therapy, with an increased RR for anthracycline-containing regimens over nonanthracycline chemotherapy, while no clear differences in survival were seen (A'Hern *et al*, 1993; Fossati *et al*, 1998).

In the adjuvant setting, anthracycline-containing regimens showed a significant benefit in relapse-free and overall survival (OS) as compared to cyclophosphamide plus methotrexate plus 5-fluorouracil (CMF) (Early Breast Cancer Trialists' Collaborative Group, 1998). Therefore, the National Institute of Health (NIH) and the St. Gallen International Consensus Panel on the Treatment of Early Breast Cancer recommended adjuvant chemotherapy, particularly with anthracyclines, for most women with early breast cancer, regardless of nodal, menopausal or hormonal receptor status (Eifel *et al*, 2001; Goldhirsch *et al*, 2001); as a consequence anthracycline-containing regimens are used as adjuvant therapy in the majority of patients with early breast cancer.

The relationship between the activity of first-line chemotherapy for metastatic breast cancer and prior adjuvant treatment is controversial, since some studies demonstrated a poorer outcome (Chauvin *et al*, 1990; Bonneterre and Mercier, 1993; Venturini *et al*, 1996; Pierga *et al*, 2001) whereas others showed an outcome similar to patients who had not received prior adjuvant chemotherapy (Buzdar *et al*, 1981; Bitran *et al*, 1983; Valagussa *et al*, 1986; Kardinal *et al*, 1988).

The objective of the present analysis was to evaluate the prognostic impact of adjuvant chemotherapy with anthracyclines on objective response rates, progression-free survival (PFS) and OS of metastatic breast cancer patients treated with epirubicin/paclitaxel (ET) regimens at the time of recurrence.

## MATERIAL AND METHODS

### Selection of patients

The patients included in this analysis were selected from five consecutive prospective studies in metastatic breast cancer coordinated by our institution; of these, four were phase II studies and one was a randomised trial.

In all studies, the treatment under evaluation had to be a combination of epirubicin and paclitaxel, and only patients allocated to this treatment were included in the present analysis. The chemotherapy regimens administered in each study and the number of patients entered on each protocol are listed in Table 1

**Table 1:** Treatment protocols and regimens

**Study**	**Phase**	**Treatment schedule**
ET	II	Epirubicin 90 mg m^−2^ day 1+paclitaxel 135–225 mg m^−2^ day 1, q.3 weeks, × 6/8 cycles
GET	II	Gemcitabine 1000 mg m^−2^ days 1 and 8+Epirubicin 90 mg m^−2^ day 1+paclitaxel 175 mg m^−2^ day 1, q.3 weeks, × 6/8 cycles
GET	II multicenter	Gemcitabine 1000 mg m^−2^ days 1 and 8+Epirubicin 90 mg m^−2^ day 1+paclitaxel 175 mg m^−2^ day 1, q.3 weeks, × 6/8 cycles
MANTA	II	Epirubicin 90 mg m^−2^ day 1+Paclitaxel 175 mg m^−2^ day 1, q.3 weeks × 8 cycles
MIG 6	III	Epirubicin 90 mg m^−2^ day 1+paclitaxel 200 mg m^−2^ day 1, q.3 weeks × 8 cycles *vs* epirubicin 120 mg m^−2^ day 1 × 4 cycles followed by Paclitaxel 225 mg m^−2^ day 1 × 4 cycles, q.3 weeks
Total no of patients	291	

ET=epirubicin/paditaxel;

GET=gemcitabine plus epirubicin plus taxel.

. Eligibility criteria were similar in the five studies. Patients were required to have histologically confirmed breast cancer and metastatic disease, with at least one bidimensionally measurable lesion, previously untreated with chemotherapy for metastatic disease. Prior adjuvant CMF was allowed if stopped at least 6 months before study entry; prior adjuvant anthracyclines were permitted if the total cumulative dose was less than 280 mg m^−2^ in case of doxorubicin and 360 mg m^−2^ in case of epirubicin and if stopped at least 12 months before study entry; prior adjuvant taxanes were not allowed. Other eligibility criteria included age less than 75 years, Eastern Cooperative Oncology Group Performance Status (ECOG–PS) ⩽2 and left ventricular ejection fraction (L-VEF) ⩾50% at bidimensional echocardiography. Prior endocrine treatment was allowed. Tumour responses were assessed according to the World Health Organisation (WHO) criteria (Miller *et al*, 1981). In all studies, cardiac evaluation was performed at baseline by physical examination, chest radiograph, 12-lead ECG and detection of L-VEF by bidimensionally echocardiography. Subsequent cardiac evaluation consisted of physical examination, and ECG recording every cycle, and echocardiography monitoring for L-VEF determination every two cycles during treatment, after the last course of chemotherapy, and at 3-month intervals during the follow-up period. The diagnostic criteria for CHF were new onset of dyspnoea, presence of peripheral oedema, cardiac enlargement or pulmonary congestion on chest radiograph, or pulmonary rales at auscultation. At the occurrence of clinical symptoms of CHF, reduced L-VEF contributed to diagnosis, and its assessment was performed by bidimensional echography. Clinical cardiotoxicity was graded according to the New York Heart Association clinical criteria for cardiac failure (Criteria Committee of the New York Heart Association: Nomenclature and Criteria for Diagnosis of Diseases of the Heart and Great Vessels (ed 8), 1979). The cumulative probability of developing cardiac toxicity was computed according to the life-table method, as reported previously (Gennari *et al*, 1999).

Results from these studies, as well as an analysis on the cardiac safety of the ET combination, have been reported individually (Conte *et al*, 1997, 2001a, 2001b; Gennari *et al*, 1999; Baldini *et al*, 2002) and all the data have been prospectively collected at the Trial Office of Pisa Department of Medical Oncology (ET and gemcitabine plus epirubicin plus taxol (GET) trials) or at the Trial Centre of the National Cancer Research Institute of Genoa (Manta and MIG 6 trials).

The present analyses were conducted on the database resulting from the pooling of the original databases of the individual studies that included 369 patients. In all, 78 patients with metastatic disease at diagnosis were excluded, leaving 291 patients for the present analyses. Of these patients, 10 were enrolled in the ET study after its publication.

### Statistical methods

The primary aim of the analysis was to assess the effect of previous adjuvant chemotherapy, with or without anthracyclines, on overall RR, CRR, PFS and OS.

All. analyses were conducted according to the intention to treat principle, in that all eligible patients prospectively enrolled in the individual studies were considered.

Univariate analyses used standard statistical methods, such as the *χ*^2^ test for comparison of proportions and the Kaplan–Meier estimator and the log-rank test for the estimation and comparison of survival curves.

The probabilities of overall response and of CR were also modelled in two separate multivariate logistic regression models as a function of the following covariates: age, disease-free interval (DFI), metastatic site, receptor status, nodal status, type of adjuvant treatment. Cox's proportional-hazard multivariate model was similarly used to assess the prognostic impact of previous adjuvant therapies on PFS and OS, while adjusting for the same covariates. All multivariate analyses used a step-down procedure based on the likelihood ratio test (Cox, 1958, 1972).

## RESULTS

### Patients' characteristics

A total of 291 patients were enrolled in the five consecutive protocols (Table 1). Of these, 101 patients (35%) were chemonaive and 190 (65%) received prior adjuvant chemotherapy, consisting of CMF in 109 patients (37%) and an anthracycline-containing regimen in 81 patients (28%). In particular, all the patients included in this analysis received the combination of 5-fluorouracil 600 mg m^−2^, epirubicin 60 mg m^−2^ and cyclophosphamide 600 mg m^−2^ for six courses as adjuvant anthracycline-containing treatment. The median age at study entry was 55 years (range 30–73 years); 72% of women less than 60 years of age had received adjuvant chemotherapy *vs* 49% of women older than 60 years. The median disease-free survival (from diagnosis of the primary tumour to distant recurrence) was 57 months (range 1–271 months). The distribution of disease-free survival among patients who had received chemotherapy was as expected, with peaks between the 2nd and the 3rd year. A different distribution in DFI was observed according to adjuvant treatment: among the 101 chemonaive patients, 14 (13.9%) had a DFI ⩽1 year as compared to one (0.9%) and two (2.5%) in CMF and anthracycline-pretreated patients, respectively. Conversely, chemonaive patients were over-represented among long-term disease free survivors as well. 11% Of the 291 patients who achieved a response to first-line ET, received high-dose consolidation treatment with peripheral blood stem cell support and 15% received prior hormonal therapy with or without radiotherapy for metastatic disease. Patients' characteristics by prior adjuvant treatment are listed in Table 2

**Table 2:** Patients characteristics

			**Type of adjuvant chemotherapy**					
	**All patients**		**None**		**CMF**		**Anthra-based**	
**Characteristic No. of patients**	**291**		**101**		**109**		**81**	
	No	**(%)**	**No**	**(%)**	**No**	**(%)**	**No**	**(%)**
*Age (years)*								
<50	103	(35.4)	24	(23.8)	48	(44.0)	31	(38.3)
51–60	104	(35.7)	34	(33.7)	42	(38.5)	28	(34.6)
61–73	84	(28.9)	43	(42.6)	19	(17.4)	22	(27.2)

*Performance status*								
0	255	(87.6)	85	(84.2)	97	(89.0)	73	(90.1)
1	31	(10.7)	14	(13.9)	11	(10.1)	6	(7.4)
>2	5	(1.7)	2	(1.9)	1	(0.9)	2	(2.5)

*Oestrogen receptor status*								
Positive	150	(51.5)	52	(51.5)	57	(52.3)	41	(50.6)
Negative	69	(23.7)	16	(15.8)	32	(29.4)	21	(25.9)
Unknown	72	(24.8)	33	(32.7)	20	(18.3)	19	(23.5)

*Axillary nodes*								
Positive	136	(46.7)	20	(19.8)	72	(66.1)	44	(54.3)
Negative	75	(25.8)	53	(52.5)	8	(7.3)	14	(17.3)
Unknown	80	(27.5)	28	(27.7)	29	(26.6)	23	(28.4)

DFI								
1 year	17	(5.8)	14	(13.9)	1	(0.9)	2	(2.5)
1 years to 3 years	112	(38.5)	19	(18.8)	52	(47.7)	41	(50.6)
3 years to 5 years	62	(21.3)	21	(20.8)	24	(22.0)	17	(21.0)
>5 years	100	(34.4)	47	(46.5)	32	(29.4)	21	(25.9)

*Dominant metastatic site*								
Viscera	206	(70.8)	65	(64.4)	80	(73.4)	61	(75.3)
Soft tissues	51	(17.5)	18	(17.8)	18	(16.5)	15	(18.5)
Bone	34	(11.7)	18	(17.8)	11	(10.1)	5	(6.2)

HDCT	32	(10.9)	10	(9.9)	12	(11.0)	10	(12.3)
HT	44	(15.1)	16	(15.8)	15	(13.7)	13	(16.0)
Median cumulative epi, range (mg sqm^−2^)			540		540		900	
			(90–720)		(90–720)		(450–1080)	

CMF=Cyclophosplamide plus methotrexate plus 5-fluorouracil; DFI=disease-free interval.

HDCT=High Dose Chemotherapy

HT=Hormonal Therapy for Metastatic Disease

.

### Cardiac safety

Overall, symptomatic CHF (class III NYHA) occurred in 13 patients (4.5%), all showing symptoms after completion of treatment: five of them developed CHF after a cumulative epirubicin dose of 1080 mg m^−2^ (360 mg m^−2^ were administered in the adjuvant setting), equivalent to724 mg m^−2^ of doxorubicin; one after a cumulative epirubicin dose of 990 mg m^−2^ (360 mg m^−2^ in the adjuvant setting), equivalent to 663 mg m^−2^ of doxorubicin; four after a cumulative epirubicin dose of 720 mg m^−1^ (two of them received 360 mg m^−2^ as part of the adjuvant setting), equivalent to 482 mg m^−2^ of doxorubicin; one after a cumulative epirubicin doses of 630 mg m^−2^, equivalent to 422 mg m^−2^ of doxorubicin and two after a cumulative epirubicin dose of 540 mg m^−2^, equivalent to 362 mg m^−2^ of doxorubicin.

### Clinical activity

Overall, a response to first-line epirubicin plus paclitaxel was observed in 192 patients (66%) (Table 3

**Table 3:** Overall RR to first-line ET according to prior adjuvant chemotherapy

		**RR**			**Multivariate analysis**				
**Prior adjuvant chemotherapy**	**No. of patients**	**No**	**%**	**P-values**	**Odds ratio**	**95% CI**	**χ^2^**	**df**	**P-value**
None	101	69	68		1				
CMF	109	69	63		0.81	0.37–1.79			
Anthracycline	81	54	67		0.92	0.43–2.01	0.29	2	
				0.5					0.86

All patients	291	192	66						

RR=response rate; ET=epirubicin/paditaxel; df=degree of freedom; CMF=Cyclophosplamide plus methotrexate plus 5-fluorouracil.

). In univariate analysis, an association between prior adjuvant anthracyclines and overall RR was not evident: RR was 68% in patients who had not received prior adjuvant chemotherapy, 63% in patients who received prior CMF and 67% in patients who received prior anthracyclines (*P*=0.5). This observation was confirmed in a multivariate logistic regression model, with RR as the dependent variable: adjusted odds ratio for response was 0.81 (95% CI: 0.37–1.79) for CMF and 0.92 (95% CI 0.43–2.01) for anthracyclines (*χ*^2^-2df=0.29, *P*=0.86). Age of patients and DFI were significantly associated with the probability of response.

Conversely, results for CPR suggested an influence of adjuvant chemotherapy on the probability of a CR (Table 4

**Table 4:** CRR to first-line ET according to prior adjuvant chemotherapy

		**CRR**			**Multivariate analysis**				
**Prior adjuvant chemotherapy**	**No. of patients**	**No**	**%**	***P*-value**	**Odds ratio**	**95% CI**	**χ^2^**	**df**	***P*-value**
None	101	22	22		1 (ref)				
CMF	109	15	14		0.40	0.17–0.98			
Anthracycline	81	11	14		0.39	0.17–0.86	5.45	2	
				0.2					0.036
All patients	291	48	16						

CRR=Complete response rate; ET=epirubicin/paclitaxel; df=degree of freedom; CMF=Cyclophosplamide plus methotrexate plus 5-fluorouracil.

). In fact, in univariate analysis, an increased probability of achieving a CR to first-line ET was observed among chemonaive patients, even though the difference was not statistically significant (chemonaive 22% *vs* CMF 14% *vs* prior anthracyclines 14%, *P*=0.2). However, in multivariate analysis, a significant association between adjuvant chemotherapy and probability of CR was seen in the final model, where age and site, besides adjuvant chemotherapy, were the only significant factors included in the equation. The model indicated that, once age and site were adjusted for, the relative odds of obtaining a CR in patients who had received adjuvant CMF or anthracyclines were 0.40 and 0.39 as compared to chemonaive patients (*P*=0.036). It was worthy noting that the probability of CR was strongly correlated with age, and no CR was observed among the 41 patients older than 60 years who had received adjuvant chemotherapy, *vs* six out of 43 (14%) chemonaive patients.

### Progression-free survival (Table 5)

**Table 5:** PFS after-first-line ET according to prior adjuvant chemotherapy

				**Multivariate Analysis**				
**Prior adjuvant chemotherapy**	**No. of patients**	**Median PFS months (95% CI)**	***P-*value**	**Hazard ratio**	**95% CI**	**χ^2^**	**df**	**P-Value**
None	101	12.5 (9.3-15.8)		1 (ref)				
CMF	109	11.0 (9.1-12.9)		1.21	0.78–1.86			
Anthracycline	81	10.2 (8.2-12.1)		1.42	0.91–2.20	2.53	2	
			0.33					0.28
All patients	291	11.2 (8.2-15.8)						

PFS=Progression-free survival; ET=epirubicin/paclitaxel; CI=Confidence interval; df=degree of freedom; CMF=Cyclophosplamide plus methotrexate plus 5-fluorouracil.

**Figure 1:**
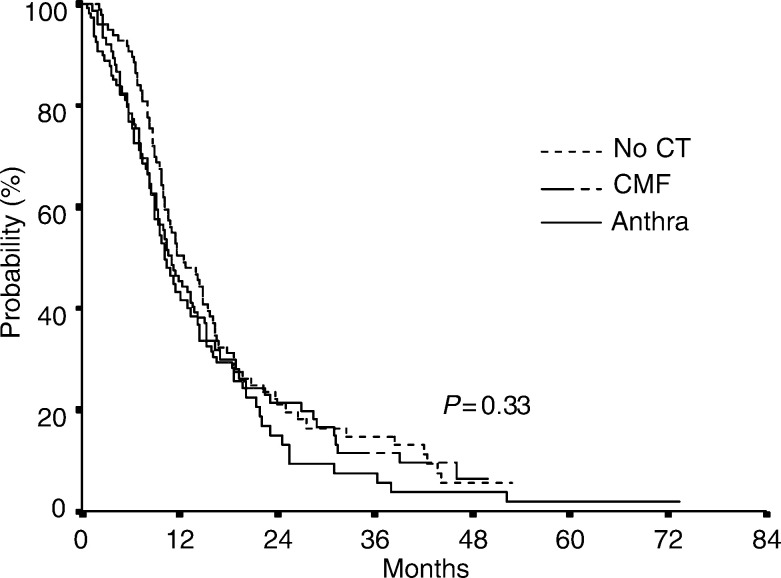
Kaplan–Meier estimate of PES by prior adjuvant therapy.

The median PFS after first-line epirubicin plus paclitaxel was 11.2. months (range 8.2–15.8 months). In univariate analysis prior adjuvant treatments failed to show relevant effects on PFS: median PFS was 12.5 months in patients who had not received prior adjuvant chemotherapy, 11.0 months in patients who received prior CMF and 10.2 months in patients who received prior anthracyclines (*P*=0.33) (Figure 1

). In multivariate Cox's proportional-hazard model, a nonsignificant increase in the risk of progression was seen in patients treated with adjuvant CMF (HR 1.21, 95% CI 0.78–1.86) or anthracyclines (HR 1.42, 95% CI 0.91–2.20) (*χ*^2^-2 df=2.53, *P*=0.28). DFI was the only factor significantly associated with progression-free survival.

### Overall survival (Table 6)

**Table 6:** OS after first-line ET according to prior adjuvant chemotherapy

				**Multivariate analysis**				
**Prior adjuvant chemotherapy**	**No. of patients**	**Median OS months (95% CI)**	***P*-value**	**Hazard ratio**	**95% CI**	***χ*^2^**	**df**	***P*-value**
None	101	27.5 (19.9–35.1)		1 (ref)				
CMF	109	23.8 (19.1–28.5)		1.16	0.68–1.99			
Anthracycline	81	20.2 (16.4–24.0)		1.35	0.78–2.33	1.24	2	
			0.61					0.54
								
All patients	291	24.1 (16.4–35.1)						

OS=overall survival; ET=epirubicin/paclitaxel; CI=Confidence interval; df=degree of freedom; CMF=Cyclophosplamide plus methotrexate plus 5-fluorouracil.

**Figure 2:**
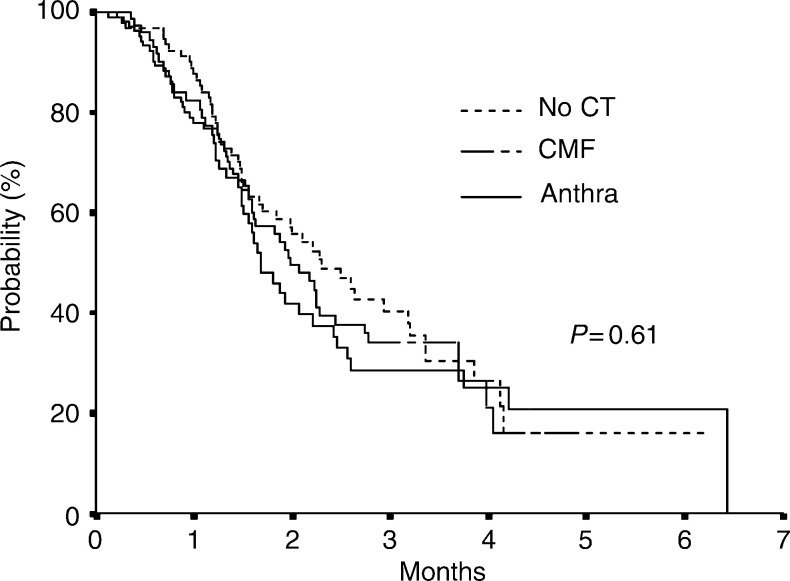
Kaplan–Meier estimate of OS by prior adjuvant therapy.

The median OS after first-line epirubicin plus paclitaxel was 24.1. months (range 16.4–35.1 months), and it was 27.5 months in patients who had not received prior adjuvant chemotherapy, 23.8 months in patients who received prior CMF and 20.2 months in patients who received prior anthracyclines; however, this difference was not statistically significant (*P*=0.61) (Figure 2

). The same, nonsignificant, association was seen in multivariate analysis, for CMF (HR 1.16, 95% CI 0.68–1.99) and anthracyclines (HR 1.35, 95% CI 0.78–2.33) (*χ*^2^-2df=1.24, *P*=0.54). Age and DFI were significantly associated with survival.

## DISCUSSION

The correlation between the efficacy of chemotherapy at the time of relapse and the previous use of adjuvant chemotherapy is still controversial. Poorer outcomes were not consistently observed in metastatic breast cancer patients previously treated with adjuvant chemotherapy (Buzdar *et al*, 1981; Bitran *et al*, 1983; Valagussa *et al*, 1986; Kardinal *et al*, 1988), although a few studies reported a detrimental effect (Chauvin *et al*, 1990; Bonneterre and Mercier, 1993; Venturini *et al*, 1996; Pierga *et al*, 2001). The present analysis was aimed at assessing the impact of adjuvant chemotherapy with anthracyclines on the outcome of 291 patients treated with the combination of epirubicin and paclitaxel at the time of relapse. Patients included in this analysis were prospectively enrolled in five controlled trials and treated over a period of five years. Unlike most previous studies, a large proportion (two of three) of our patients had received adjuvant chemotherapy, with a sizeable proportion receiving an anthracycline-containing regimen. As a consequence, this study can better assess the effect of previous anthracyclines on the probability of response and prognosis in patients with metastatic disease undergoing modern first-line chemotherapy regimens using epioublicin and Paclitaxel However, due to the relatively low dose of prior anthracycline allowed (epirubicin <360 mg m^−2^ and doxorubicin <280 mg m^−2^), the results of this analysis might be applicable only to this specific subset of patients.

Furthermore, the patients included in this analysis were prospectively enrolled, treated and followed in five phase II and III clinical trials, and a significant selection bias can be ruled out, as only patients with metastatic disease at diagnosis were excluded. The main limitation of our study is its small size, which precludes statistically stable estimates and firm clinical conclusions, especially in the light of the potential confounding effect of various factors, some of which are indeed associated with the use of chemotherapy (eg age at diagnosis) and/or could not be accurately assessed in all patients at the time of diagnosis of metastatic disease (pathological and biological factors).

Our results confirm the high level of activity of the administration of epirubicin and paclitaxel, either in combination or sequence, with an overall RR of 66%. This might be due to the introduction of a new active agent such as paclitaxel as well as to a better selection of patients potentially responsive to chemotherapy. Furthermore, adjuvant chemotherapy with or without anthracyclines does not seem to affect the probability of achieving a response to first-line ET. An intriguing finding was the negative association between the probability of CR and previous chemotherapy, which in multivariate analysis achieved statistical significance. It is worth noting that no CRs were observed in patients older than 60 years previously treated with adjuvant chemotherapy. Yet, this negative effect appeared to be similar in patients treated with adjuvant anthracyclines or CMF, since the odds of obtaining a CR was reduced by 60% in both CMF- and anthracyclines-pretreated patients compared to chemonaive patients.

While no effect of previous chemotherapy on PFS was seen, there was some indication of a shorter survival in patients who had received adjuvant chemotherapy, and particularly anthracyclines-containing regimens, even though the difference fell short of statistical significance. In fact by multivariate analysis the probability of a poorer outcome was increased by 40% in terms of PFS and by 35% in terms of OS. This finding could be interpreted in the light of the reduced CRR observed in patients who received adjuvant chemotherapy (A'Hern *et al*, 1993). As a matter of fact, if only the small group of patients failing to achieve a complete response as a consequence of adjuvant chemotherapy experienced a detrimental effect on survival, this difference would be diluted when the entire groups of patients are compared.

In accordance with previously reported studies (Bonneterre and Mercier, 1993; Venturini *et al*, 1996; Pierga *et al*, 2001), in our series, the risk of progression and death was increased in pretreated patients, and particularly in those who received an anthracycline in the adjuvant setting. However, when indirect comparisons were performed, the magnitude of this difference was minor of those reported previously, and did not reach statistical significance. The inconsistency between our results and those of previous analyses that suggested a negative prognostic impact of adjuvant treatments could be attributed to a better selection of the patients as well as to the regimens employed in our studies, and particularly to the activity of taxanes in metastatic breast cancer (Ghersi *et al*, 2003).

With only 13 episodes of symptomatic CHF, anthracycline-induced cardiotoxicity was not an issue in this analysis. Moreover, in accordance with our previous report (Gennari *et al*, 1999), the low incidence of CHF, up to cumulative epirubicin doses of 990 mg m^−2^, suggests that the ET regimen might be safely administered even in case of prior adjuvant epirubicin.

Overall, this analysis confirms that modern chemotherapy regimens, including epirubicin and paclitaxel, provide satisfactory results in metastatic breast cancer patients, regardless of previous adjuvant chemotherapy. Conversely, it suggests that the probability of CR is decreased in previously treated patients and particularly in women older than 60 years, even though the consequences of this decrease on survival are not clear.

These findings do not provide direct evidence on the efficacy and the importance of anthracyclines in chemotherapy regimens administered to breast cancer patients relapsed after adjuvant anthracyclines. Accordingly, the possibility that the same results could be obtained with different regimens without cardiac toxicity cannot ruled out. However, the high level of activity observed in previously treated patients (>60%) can hardly be attributed entirely to paclitaxel, which in monochemotherapy is associated with a RR ranging from 26 to 47% in anthracycline-pretreated patients (Gianni *et al*, 1994; Fountzilas *et al*, 1996; Donadio *et al*, 2001).

In conclusion, on the basis of these results, hormone negative or refractory metastatic breast cancer patients should be treated with first-line chemotherapy, including the most active agents, irrespective of prior adjuvant treatment.
